# The role of GABA in islet function

**DOI:** 10.3389/fendo.2022.972115

**Published:** 2022-09-29

**Authors:** D. Walker Hagan, Sandra M. Ferreira, Gustavo J. Santos, Edward A. Phelps

**Affiliations:** ^1^ J. Crayton Pruitt Family Department of Biomedical Engineering, University of Florida, Gainesville, FL, United States; ^2^ Islet Biology and Metabolism Lab – I.B.M. Lab, Department of Physiological Sciences, Center of Biological Sciences, Federal University of Santa Catarina - UFSC, Florianópolis, Brazil

**Keywords:** γ-Aminobutyric acid (GABA), islet, pancreas, signaling, receptor, insulin, beta cell

## Abstract

Gamma aminobutyric acid (GABA) is a non-proteinogenic amino acid and neurotransmitter that is produced in the islet at levels as high as in the brain. GABA is synthesized by the enzyme glutamic acid decarboxylase (GAD), of which the 65 kDa isoform (GAD65) is a major autoantigen in type 1 diabetes. Originally described to be released *via* synaptic-like microvesicles or from insulin secretory vesicles, beta cells are now understood to release substantial quantities of GABA directly from the cytosol *via* volume-regulated anion channels (VRAC). Once released, GABA influences the activity of multiple islet cell types through ionotropic GABA_A_ receptors and metabotropic GABA_B_ receptors. GABA also interfaces with cellular metabolism and ATP production *via* the GABA shunt pathway. Beta cells become depleted of GABA in type 1 diabetes (in remaining beta cells) and type 2 diabetes, suggesting that loss or reduction of islet GABA correlates with diabetes pathogenesis and may contribute to dysfunction of alpha, beta, and delta cells in diabetic individuals. While the function of GABA in the nervous system is well-understood, the description of the islet GABA system is clouded by differing reports describing multiple secretion pathways and effector functions. This review will discuss and attempt to unify the major experimental results from over 40 years of literature characterizing the role of GABA in the islet.

## Introduction

**Figure 1:**
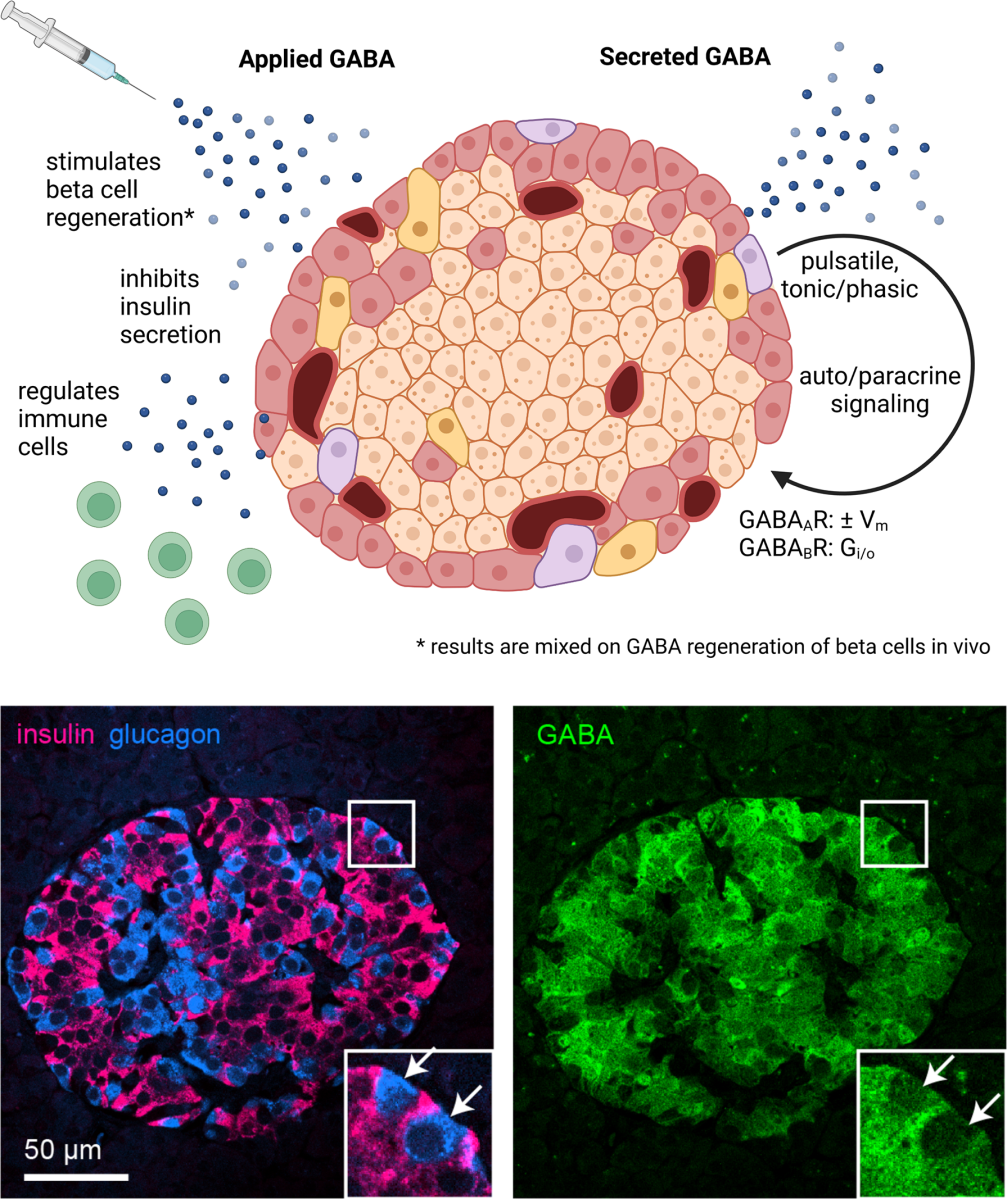
GABA in the whole islet. Application of exogenous GABA has various effects on the islet including stimulation of beta cell regeneration, inhibition of insulin secretion, and negative regulation of immune cells. Endogenous GABA levels are highly enriched in the islet, as high as in the brain, and GABA is synthesized in and secreted from the beta cells. Immunofluorescence image depicts a human islet. GABA is secreted *via* multiple pathways that are both regulated and unregulated by glucose and with pulsatile, tonic, or phasic dynamics. Once secreted, GABA acts *via* GABA_A_R ligand-gated chloride channels and GABA_A_R inhibitory G protein coupled receptors. Set by the chloride equilibrium potential, in beta cells GABA_A_R signaling can be excitatory in low glucose and inhibitory in high glucose, while in alpha cells GABA_A_R signaling is inhibitory. GABA_B_R signaling is also inhibitory but may only be active in mouse and not human beta cells under typical physiological conditions. Created with BioRender.com.

Gamma-aminobutyric acid (GABA) is the major inhibitory neurotransmitter in the mammalian central nervous system (CNS) where it serves to regulate neuronal excitability. Outside of the CNS, high concentrations of GABA are found within the insulin-producing beta cells of the pancreas (**Figure 1**) (1). Glutamic acid decarboxylase 65 (GAD65; *Gad2*), one of the two mammalian enzymes that synthesize GABA from glutamate (the other being GAD67, *Gad1*), is expressed in human beta cells, and is a major autoantigen in type 1 diabetes (T1D) (2). According to single-cell RNA sequencing datasets, the expression of *Gad1* or *Gad2* in neural and pancreatic endocrine cells is at least an order of magnitude higher than in any other cell type (Human Protein Atlas proteinatlas.org, Tabular Muris czbiohub.org/tabula-muris/) (3–5). Thus, the ability to synthesize large amounts of GABA *via* the GAD enzyme is essentially unique to neural and beta cells with the caveat that GABA has recently been shown to be synthesized by glial cells (6) and B cells (7). GABA concentrations have been measured to be ~40 μmol/g in brain tissue and ~20 μmol/g in the islets, while GABA in other tissues is typically below 1 μmol/g (7, 8). Intracellular GABA concentration from islet lysate is estimated to be 4 mM (9) while interstitial GABA levels in the islets are measured in the range of 10 nM to 10 μM (10–12). Several types of immune cells express receptors for and functionally respond to GABA (13, 14). GABA is also present in the adrenal glands, testes, prostate, kidney, gastrointestinal track, oviduct, placenta, uterus, lymph nodes, and spleen among other tissues (15). Low-level GAD expression, alternative GABA biosynthesis pathways, uptake of GABA from circulation, dietary sources, or production by microbiota may be important for GABA’s diverse functions throughout the body (15–17).

Many roles for GABA in the islet have been proposed and studied for decades, but the overall importance of GABA to islet function has not been completely elucidated (18, 19). Surprisingly, a beta cell-conditional knockout mouse for GABA biosynthesis that would provide clear evidence supporting the specific biological role of GABA in the islet has never been generated. Considering that the islet uniquely invests in high levels of GABA biosynthesis at the risk of invoking GAD autoimmunity, it strikes us as intuitive that GABA must be of central importance for normal islet physiology.

GABA’s behavior as a neurotransmitter in the islet is perhaps the most widely studied role for which there is abundant data supporting auto- and paracrine signaling within and between islet endocrine cells. The evidence varies depending on experimental conditions as to whether GABA is stimulatory or inhibitory toward secretion of major islet hormones. The auto- and paracrine action of GABA can be understood through the physiology of GABA_A_ and GABA_B_ receptors. GABA_A_ receptors (GABA_A_R) are ligand-gated chloride channels that affect membrane excitability. Whether GABA_A_R mediated Cl^-^ currents are excitatory or inhibitory is governed by intra- and extracellular chloride ion concentration, membrane potential, and transmembrane ion transport. Intracellular chloride concentration [Cl^-^]_i_ varies depending on the cell type, being higher in beta cells than in alpha cells or neurons. GABA also signals through GABA_B_Rs, which are inhibitory G protein coupled receptors that open G protein-coupled inwardly-rectifying potassium channels (GIRKs), inactivate some types of voltage-gated Ca^2+^ channels, and reduce the activity of adenylyl cyclase reducing cAMP. High levels of intracellular GABA contribute to the unique beta cell metabolism where GABA feeds into the tricarboxylic acid (TCA) cycle *via* the GABA shunt (20). The combination of GABA_A_R, GABA_B_R, and GABA metabolism contribute within a symphony of other signaling inputs to shape pancreatic islet hormone release (21).

Beyond its auto/paracrine and metabolic roles, exogenously delivered GABA has been reported to be a beta cell trophic factor, preserving and/or regenerating beta cell mass (22, 23). The islet regenerative capacity of GABA has been particularly studied in situations of T1D or beta cell depletion (23–26) where GABA appears to activate or contribute to transdifferentiation of alpha cells into beta cells in insulin-deficient or diabetic mice (22, 27). However, the potential for GABA to directly and substantially regenerate beta cells *in vivo* should be treated with caution due to other studies that produced negative results in mice and non-human primates (28–31). Finally, GABA has immunomodulatory effects on immune cells throughout the body which express all the components for GABA signaling (32). GABA affects immune cell activation, migration, cytokine secretion, and cytotoxic responses and promotes monocyte differentiation into anti-inflammatory phenotypes (7, 32, 33). In the context of autoimmunity, GABA appears to be anti-inflammatory in T1D (24, 33, 34) and experimental autoimmune encephalomyelitis (EAE) (35), suggesting that GABA could be a mechanism for islet immune protection. While further discussion of the beta cell regenerative and immune regulatory aspects of GABA are topics of keen interest, this review focuses primarily on the signaling and metabolic aspects of GABA in the islet.

## Identification of GABA in the pancreas

The first measurements of amino acid concentrations in pancreas tissue were performed as early as the 1950s. The concentration of GABA in whole pancreas and other tissues was low enough to indicate the brain as the only organ maintaining a significant concentration of GABA (36). It wasn’t until 1972 that scientists from Sweden investigated amino acid concentrations in the pancreatic islet, indicating a measurable presence of GABA in islets with no detection in exocrine tissue (37). Simultaneously, as islet biology was gaining traction, a group in Japan hypothesized that islets would have heavy innervation and, therefore, high concentrations of GABA. Manually extracting islets from frozen rat cryosections and analyzing the GABA content, Okada et al. found the concentration of GABA within the islet to match the concentrations in neurons (1). This group next measured GAD activity and GABA concentrations in human insulinoma, confirming that human beta cells express GAD and synthesize GABA (8). As insulinoma has a much higher concentration of beta cells than innervation, this result implicated the beta cells themselves as synthesizing GABA.

Back in Sweden, Gylfe and Sehlin studied metabolic pathways in amino acid synthesis in the ob/ob mouse, a mouse that models type 2 diabetes (T2D). The addition of high glucose along with leucine increased the concentration of GABA in the islet, confirming a potential link between GABA synthesis and the glucose-sensing function of the islet (38). Their results directly implicated beta cells as the main islet cell type to synthesize GABA. Following the discovery that islet beta cells synthesize GABA, Taniguchi et al. in Japan treated rats with streptozotocin to eliminate the beta cells, resulting in islet GABA concentrations as low as acinar tissue. In addition, histology showed that nerve terminals remained intact, proving that the source of islet GABA is primarily from the beta cells (39, 40). To determine whether islets utilize GABA similarly to neurons, radiolabeled 3H-GABA uptake studies by Okada et al. were performed on chopped pancreas tissue and sections of substantia nigra (41). Pancreas 3H-GABA uptake was much slower in islets than that of the substantia nigra, indicating that pancreas differs in GABA reuptake mechanisms from neural tissue. Interestingly, autoradiographic imaging of the 3H-GABA uptake in rat pancreas show that high-affinity GABA uptake does occur but only in a subpopulation of the somatostatin-positive delta cells (42, 43). These early 3H-GABA uptake results have garnered relatively few citations since they were published more than 30 years ago, and have not been confirmed in human islets, but are of keen interest because they suggest that delta cells may be important to clear excess interstitial GABA in the islet in a manner analogous to astrocytes in the CNS. Decades later, Menegaz et al. showed that human beta cells do not express detectable levels of any of the four membrane GABA transporters (GAT1, *Slc6a1*; GAT2, *Slc6a13*; GAT3, *Slc6a11*; and BGT1, *Slc6a12*) (12, 44). Both endocrine and exocrine cells of the pancreas do express the low-affinity GABA-permeable taurine transporter TAUT (*Slc6a6*), which explains the overall lower rate GABA reuptake in the pancreas compared to brain tissue and could account for a low basal level of GABA reuptake. Modern single-cell RNA-seq data shows that a subpopulation of delta cells may express the high-affinity GABA transporter GAT3 (*Slc6a11*) (Human Protein Atlas proteinatlas.org, Tabular Muris czbiohub.org/tabula-muris/) (3–5) which could explain the specific delta cell 3H-GABA uptake. Whether GABA reuptake by delta cells represents a metabolic coupling to beta cells, a GABA degradation mechanism, or repackaging of GABA in preparation for re-secretion is unknown.

Islet GABA content is reportedly significantly reduced in human islets from donors with T2D (10, 12, 45) and depleted in islets from donors with T1D, certainly due to the overall reduction in beta cells, but GABA is also depleted in the remaining beta cells (12). The mechanism that drives the reduction in islet GABA during diabetes is unknown. Functional consequences of diabetes-related loss of islet GABA have not been studied *in vivo*. Pharmacological studies that manipulate GABA synthesis and catabolism or GABA receptor signaling, show that loss of GABAergic input profoundly impacts glucose-responsive insulin and glucagon secretion *in vitro* (12, 46, 47). Islets from donors with T2D attempt to compensate for reduction in GABA by increasing the affinity of GABA_A_R, possibly by expressing higher affinity receptor subunits (10).

## GABA subcellular localization and biosynthesis

### GABA synthesis by GAD

GABA is synthesized by the enzyme GAD from the precursor glutamic acid, a non-essential amino acid tied to both cellular metabolism and signaling (48). GAD forms a homodimeric structure, with pyroxidal phosphate (PLP), the active form of vitamin B6, a required cofactor at the binding site, creating a glutamate-PLP complex. This complex naturally favors the carbanion intermediate, ultimately resulting in decarboxylation of glutamate to generate GABA, releasing a molecule of CO_2_ in the process (48). GAD exists in two isoforms: GAD67 and GAD65, with molecular weights of 67 kDa and 65 kDa, respectively. These two isoforms catalyze the same decarboxylase reaction, but fulfill different functional roles, are expressed from different genes, synthesize GABA at different locations in the cell, and are differentially regulated (49).

Beta cells express the two isoforms of GAD differently depending on the species. Human beta cells only express GAD65, while mouse beta cells express almost entirely GAD67. Rat beta cells express both GAD65 and GAD67 (50). Whether species differences in GAD isoform expression result in functional differences in the islet GABA system has not yet been experimentally addressed.

### GAD protein trafficking in beta cells

**Figure 2:**
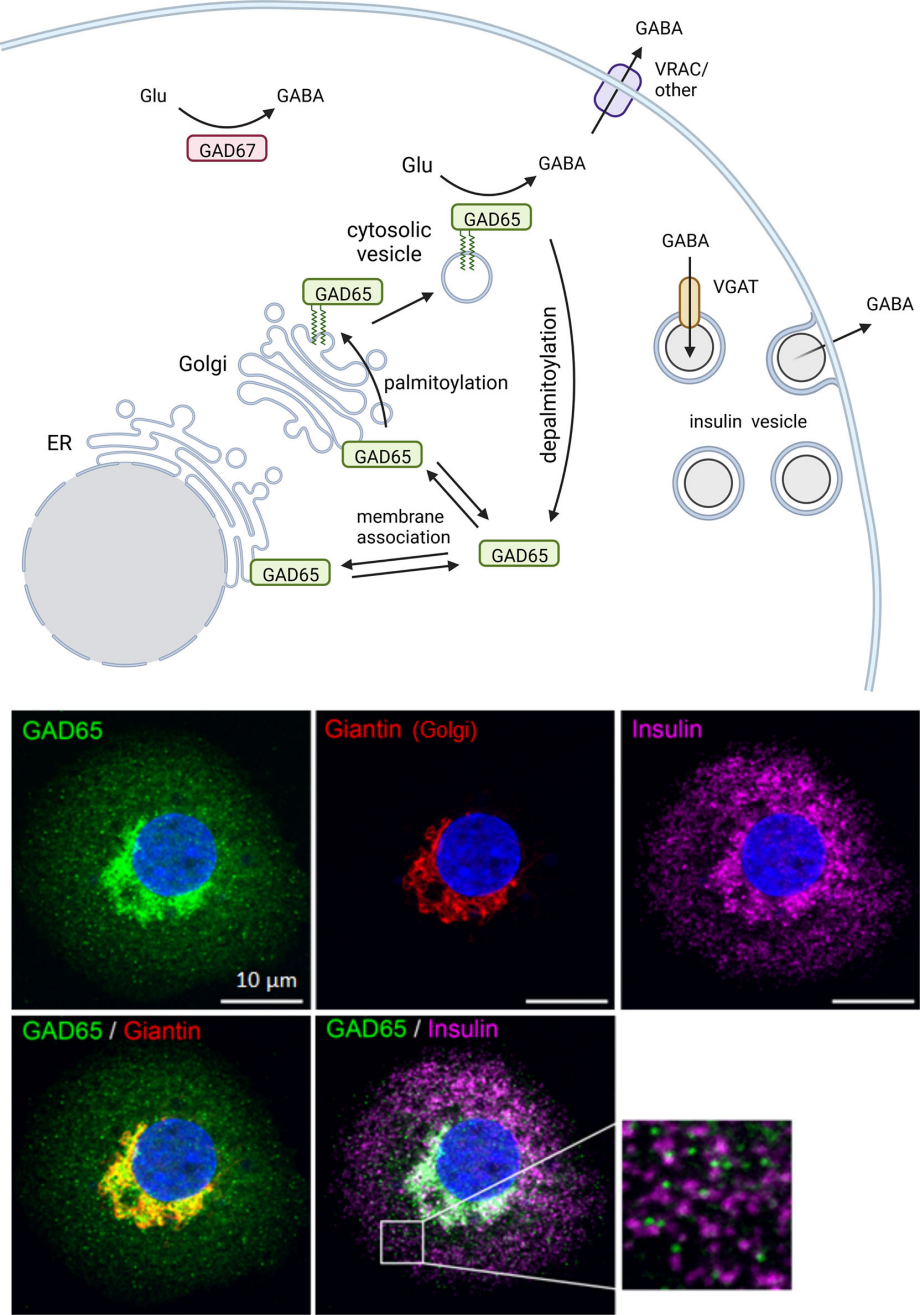
Intracellular trafficking pattern of GAD in beta cells. The two GABA-synthesizing enzymes, GAD65 and GAD67, are synthesized as cytosolic proteins. GAD65 becomes associated with the cytosolic face of ER and Golgi membranes and subsequently modified by double palmitoylation which allows forward trafficking onto post-Golgi intracellular vesicles. The palmitoylation step is reversible and serves to regulate localization of GABA synthesis between cytosolic and membrane-anchored pools of enzyme. GAD67 is the predominant isoform expressed in mouse beta cells while only GAD65 is expressed in human beta cells. In human beta cells GAD65 localizes to Golgi membranes and cytosolic vesicles that are distinct from insulin containing vesicles. Created with BioRender.com.

Lipophilic post-translational modifications regulate GAD intracellular localization. Both GAD65 and GAD67 are synthesized as cytosolic proteins but GAD65 becomes irreversibly post-translationally modified at the N-terminal domain to hydrophobically associate with lipid membranes. Membrane-anchored GAD65 then becomes palmitoylated at cysteines 30 and 45, which results in GAD65 sorting to the trans-Golgi network and targeting to post-Golgi axonal vesicles and pre-synaptic clusters in neurons or peripheral vesicles in beta cells. The palmitoylation of GAD65 is reversible and serves to regulate the localization of GABA synthesis. GAD67, is a hydrophilic soluble molecule that does not anchor to membranes intrinsically. Therefore, the majority of membrane-bound GAD is GAD65, while GAD67 is mainly localized in the cytosol (**Figure 2**).

Both isoforms of GAD form homodimers. Dimerization is essential to GAD enzyme function as the active site occurs at the dimer interface. GAD can also form heterodimers between GAD65 and GAD67 which allows GAD67 to become membrane anchored through complexation with GAD65 or by a distinct GAD65-independent mechanism that involves association with a different membrane-anchoring moiety (51). In contrast to neurons, beta cells lack a mechanism for homodimerized GAD67 to become membrane bound. This results in different intracellular trafficking patterns and, subsequently, different localization of GAD between beta cells and neurons (51). Furthermore, the species-specific beta cell expression of GAD65 and GAD67 results in mouse beta cells expressing cytosolic GAD67 and human beta cells expressing GAD65 distributed between ER/Golgi membrane-anchored, vesicular, and cytosolic localizations.

GAD65 can function as an active holoenzyme or as an inactive apoenzyme, due to changes in affinity for the cofactor PLP. GAD67 remains active with high affinity for PLP (52). Palmitoylation-deficient mutants of GAD65 exhibit a lower ability to convert glutamate to GABA, hinting that GAD65 activity may be higher when membrane bound and palmitoylated (53). On the other hand, endoplasmic reticulum (ER) stress in beta cells induced by palmitic acid or thapsigargin disrupts the palmitoylation cycle of GAD65 resulting in a redistribution of the protein to the Golgi (54). A similar pattern of Golgi accumulation of GAD65 is observed in human islets from donors with type 2 diabetes (T2D), who are GAD autoantibody positive (GADA^+^), or in residual beta cells in islets from donors who have T1D (12, 54). It is unknown whether the Golgi redistribution affects GAD enzyme function but islet GABA content and secretion are markedly reduced in human islets from donors with diabetes.

### GAD subcellular localization suggests a nonvesicular GABA release mechanism in beta cells

Low resolution immunostaining studies of βTC3 mouse insulinoma cells published in the early 1990’s showed that GAD65 staining colocalized at the cellular level with synaptophysin, a synaptic vesicle marker (55). This early staining was interpreted within the context of known GABA secretion pathways in neurons to mean that beta cells likely secrete GABA from *synaptic-like microvesicles*, a population of small secretory vesicles proposed to function in endocrine cells as a counterpart to the synaptic vesicles in neurons. It was hypothesized that beta cells utilize the putative synaptic-like microvesicles for regulated secretion of GABA. GAD65 synthesizes GABA on the outward surface of intracellular membranes, releasing GABA into the cytosol. In GABA-producing neurons, GAD65 colocalizes with the vesicular GABA transporter (VGAT; *Slc32a1*) also called the vesicular inhibitory amino acid transporter (VIAAT) in synaptic vesicles (56). By association with GAD65, VGAT mediates transport of the product GABA into the synaptic vesicle lumen, where it accumulates in preparation for regulated secretion (48, 57). The existence of an analogous GABA-secreting system in beta cells would require co-expression of GAD65 and VGAT together with other synaptic vesicle proteins (58).

However, in human and rodent beta cells, expression of VGAT remains below detection limits for immunohistochemistry (12, 59) and single-cell RNA sequencing (Human Protein Atlas proteinatlas.org, Tabular Muris czbiohub.org/tabula-muris/) (3–5). Rather, VGAT is expressed in human delta cells and rodent alpha cells (12). Consistent with a lack of VGAT expression, most human beta cells exhibit a uniform cytosolic staining pattern for GABA, although some beta cells have vesicular GABA which colocalizes with insulin (12). Ultrastructural studies have been performed to observe subcellular localization of GABA in islet sections *via* immunogold labeling. GABA-specific immunogold particles mostly do not target any particular vesicular structure within the beta cell, instead indicating that GABA resides uniformly within the cytosol of beta cells, although some studies showed a population of GABA-containing insulin secretory vesicles (60–62).

While GABA itself is predominantly cytosolic in beta cells, the GABA-synthesizing enzyme GAD65 localizes to strongly punctate cytoplasmic structures (51), previously described to be synaptic-like microvesicles (55, 63). Because the concentration of GABA in the lumen of most GAD65^+^ puncta or insulin granules appears no different than that in the cytosol, these GAD65^+^ puncta seen in beta cells are unlikely to be synaptic-like microvesicles for GABA secretion. The caveat to cytosolic GABA is that a subpopulation of beta cells do express VGAT and thus exhibit clear vesicular GABA (12). Whether VGAT^+^ beta cells with vesicular GABA represent a form of beta cell sub-type with functional consequences or are merely a stochastic phenomenon is unknown.

## GABA in beta cell metabolism

In the classical neurotransmitter paradigm of GABA, there are two primary mechanisms for the effects of GABA: activation of GABA receptors on the plasma membrane (64) and interaction with the GABA shunt (65), affecting mitochondrial function. Beta cells metabolize GABA *via* the GABA-shunt, a catabolic pathway initiated by transamination of GABA with α-ketoglutarate by the mitochondrial enzyme GABA transaminase (GABA-T) (66). The products of the GABA transamination reaction are glutamate and succinate-semialdehyde (SSA). The generated glutamate can be converted back into GABA by GAD, while the generated SSA is rapidly oxidized by the enzyme SSA-dehydrogenase to produce succinate. This pathway is referred to as the GABA-shunt because it shunts excess α-ketoglutarate in the mitochondria directly to succinate. The GABA-shunt bypasses α-ketoglutarate dehydrogenase, the primary site of control of the metabolic flux through the TCA cycle (67). Furthermore, alpha-ketoglutarate dehydrogenase activity is significantly lower in rodent islets relative to the activity of GABA-T (20), both of which use alpha-ketoglutarate as a substrate. As a result, beta cells should be able to readily metabolize their high intracellular concentrations of GABA (12). Inhibitors of GABA-T profoundly increase GABA levels in beta cells (68) and neurons (69), indicating that GABA-shunt carries a substantial flux of carbon under normal conditions (65, 70, 71).

In rodent islets, a correlation between the metabolic state of the islet and GABA metabolism is observed (72). In beta cells, GABA may serve a role in regulating ATP synthesis, as GABA levels are inversely correlated with ATP-producing and ATP-consuming activities (72). Less GABA is released during elevated glucose metabolism and this effect is potentiated by ATP-consuming activities (72). It is attractive to hypothesize that beta cells draw on their pool of available GABA to increase carbon flux through the TCA during periods of high ATP demand. Thus, GABA-shunt is proposed to be a metabolic highway used by the beta cell to amplify the rate of ATP production (73). Such a relationship would likely entrain the rate of GABA release to beta cell metabolic oscillations (74, 75). We caution that the contribution of GABA metabolism to hormone secretion is probably minor relative to receptor-mediated GABA signaling.

## GABA secretion from beta cells

**Figure 3:**
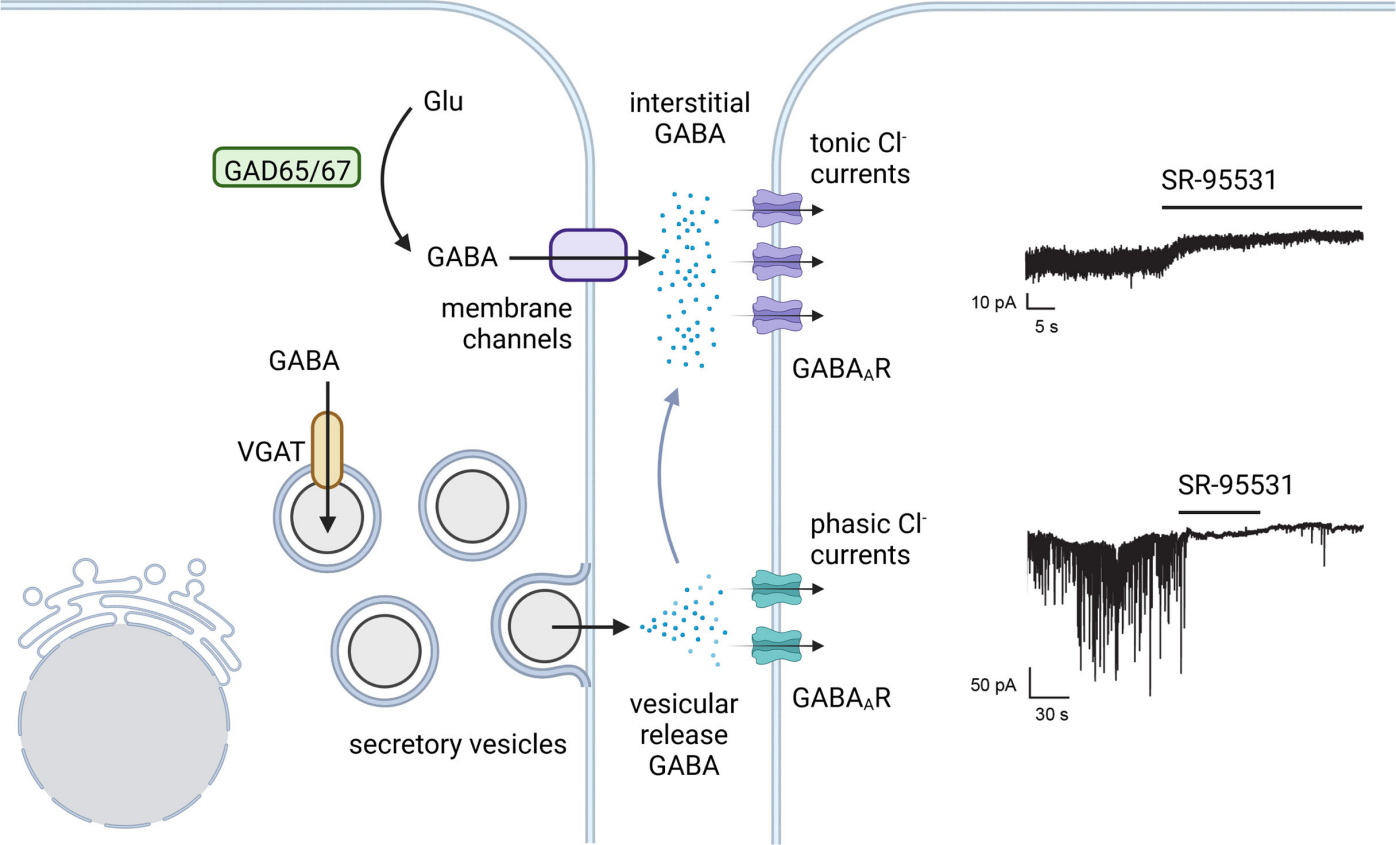
Tonic and phasic islet GABA. There is evidence favoring both a constant background secretion of GABA that is unregulated by glucose and a vesicular secretion of GABA from the insulin secretory vesicles. In analogy to synaptic and tonic GABA in neurons, vesicle released GABA in beta cells is proposed to trigger phasic Cl^-^ currents *via* low affinity GABA_A_Rs. Tonic Cl^-^currents from higher affinity GABA_A_R subtypes provide a constant current in response to continuously replenished interstitial GABA. Example GABA_A_R current traces in human beta cells from Braun et al. 2010 (76) (with permission) where both tonic and phasic currents were shown to be sensitive to the GABA_A_R antagonist SR-95531. Created with BioRender.com.

There is evidence to support both vesicular and tonic routes of GABA secretion from beta cells (**Figure 3**). A simple approach to study regulation of GABA secretion from beta cells is *via* glucose stimulation. If GABA secretion depends on glucose, it would be logical to hypothesize Ca^2+^-triggered vesicular fusion as a release mechanism. Counter to this hypothesis, macroscopic GABA secretion does not appear to be acutely regulated by glucose and is instead proportional to the availability of glutamine (20). Islet GABA release not being triggered by glucose stimulation is consistent with biochemical measurements by Baekkeskov, Caicedo and Phelps (12), Pipeleers (20, 72, 77), Tamarit-Rodriguez (70, 73, 78), Roper (79), and Naji (68). Instead of glucose, the release of GABA correlates closely with the total intracellular GABA content, indicating that GABA release is likely a facilitated process rather than active. Islet GABA release not being induced by glucose is somewhat surprising and counterintuitive, considering how different the process appears to be from how GABA is secreted by neurons.

### Vesicular GABA secretion

A series of several papers by the Rorsman group explored the question of quantized (vesicular) GABA release from islets and showed a glucose-dependent release of GABA in beta cells at the level of individual granules. This quantized GABA release can also be triggered by voltage-clamp depolarizations and by cytoplasmic Ca^2+^ infusion through patch-pipette (62, 76, 80–83). In these studies, GABA secretion was measured by overexpressing a GABA_A_R in beta cells. Endogenous GABA release events then activated these GABA_A_Rs in the same cell, giving rise to transient inward currents monitored by electrophysiology. While initially concluding to have detected individual GABA exocytotic events from synaptic-like microvesicles (83), the Rorsman group subsequently revised their conclusion and reported that quantized GABA release occurred from insulin secretory granules due to co-release with serotonin which also accumulates in insulin secretory vesicles (62, 76). However, only 13% of serotonin/insulin secretion events detected a co-release of GABA (76); and only 17% of large dense core vesicles (LDCV) were labeled with an immunogold antibody specific for GABA (62). These results suggest that only a small subpopulation of insulin secretory vesicles accumulates GABA that is co-released with insulin and regulated by glucose (76). This vesicular GABA could reflect the population of rare VGAT^+^ beta cells reported by Menegaz et al. (12). A recent examination of insulin granule subpopulations corroborates the existence of separate populations of VGAT^+^ and VGAT^-^ insulin granules (84). Alternatively, vesicular GABA accumulation could be mediated by another vesicular monoamine transporter such as vesicular monoamine transporter 2 (VMAT2; *Slc18a2*). VMAT2 is expressed in beta cells (85, 86). Although the primary substrates of VMAT2 are the monoamines dopamine, norepinephrine, serotonin, and histamine, there is evidence in neurons to suggest VMAT2 can function as a vesicular GABA transporter in the absence of VGAT (87).

### Tonic GABA secretion

Pertinent to our discussion, Rorsman and colleagues consistently reported a high rate of basal (not associated with vesicular fusion) GABA release that was unregulated by glucose or pharmacological regulators of insulin secretion (76). This basal GABA current constitutes 25% of the total internal beta cell GABA content per hour (77). Detection of basal GABA release by tonic activity of GABA_A_R expressed in GABA sniffer cells could be blocked by SR-95531 and was not associated with vesicular fusion (62, 76). This high level of non-vesicular GABA release was also independently observed by Pipeleers (77). Due to this unregulated GABA release, Rorsman and Pipeleers each independently concluded that the beta cell must be equipped with a second pathway for release of GABA from the cytosol that is non-vesicular and does not follow changes in insulin release (62). Thus, GABA and insulin must follow different routes of storage and release. However, a mechanism for non-vesicular GABA release from beta cells remained undescribed for another decade.

In 2019, Menegaz et al. published that the volume-regulated anion channel (VRAC) is a mechanism for non-vesicular release of GABA from the cytosol of beta cells (12). VRAC is an osmo-sensitive anion (Cl^-^) channel that controls cell volume. VRAC channels are hetero-hexamers complexes composed of six subunits of LRRC8 family proteins LRRC8A, B, C, D or E (88, 89). The subunit LRRC8A (also known as Swell1) is obligatory for functional VRAC channels while its heterohexamer partners LRRC8B-E confer functional heterogeneity (88, 90–92). In addition, one VRAC isoform (LRRC8A/D) has low Cl^-^ conductance and is specialized for permeability to cytosolic organic osmolytes such as GABA and taurine (88, 90–92). The expression of LRRC8A, B, and D in primary beta cells has been confirmed by rt-PCR and western blot (93–95). However, the hypothesis that GABA secretion is independent of glucose and Ca^2+^ does not take into account the known role of VRAC as a glucose sensor, as glucose-inducible VRAC Cl^-^ currents are observed in beta cells (94, 95). This discrepancy is resolved by other data showing that glucose may induce VRAC-dependent Cl^-^ currents but does not induce VRAC-dependent organic osmolyte efflux from islets (12, 78). One explanation for this may be that the signal required to activate the GABA permeant isoform of the VRAC channel (LRRC8A/D) is different than for the other Cl^-^ permeable VRAC isoforms. While large changes in osmolarity are a tool to study VRAC opening, this stimulus is unlikely to be physiological. Thus, the endogenous trigger for VRAC-dependent GABA release in beta cells remains to be discovered.

### Evidence of pulsatile GABA secretion

To understand islet GABA secretion dynamics, Menegaz et al. measured GABA release from islets in real time using biosensor cells expressing a recombinant GABA_B_R coupled to intracellular Ca^2+^ mobilization and placed in proximity to an islet in a laminar flow field. GABA release from islets was observed to follow a pulsatile pattern with a period between 4 and 10 minutes (12), similar to pulsatile insulin secretion. The pulsatile GABA release was shown to be unregulated by glucose and was not inhibited by removal of extracellular calcium, depolarization with KCl, or opening ATP-gated potassium channels with diazoxide, consistent with a non-vesicular mechanism of secretion. Inhibiting GABA biosynthesis with allyglycine acutely decreased the amplitude of GABA pulses. Likewise, inhibiting GABA catabolism with vigabatrin increased the amount of GABA released per pulse. Thus, GABA efflux is strongly coupled to the rate of GABA biosynthesis and secretion. The Roper lab independently observed the coupling of GABA secretion to islet metabolism and showed that GABA release is not triggered by high glucose (79). Genetic models to knock out or knock down LRRC8A, the obligate subunit of VRAC channels, eliminated pulsatile GABA secretion and established VRAC as a conveyer of the GABA efflux pulses (12). Whether the pulsatile pattern is produced by vesicular release, opening and closing of VRAC channels; or whether GABA membrane permeability remains relatively constant, and pulses mainly reflect oscillations in the rate of GABA biosynthesis is unknown. It is also not yet known whether GABA oscillations are coordinated with insulin secretory pulses, oscillations in cytosolic Ca^2+^, cellular metabolism, or some other rhythmic pattern in the beta cells.

It is interesting to speculate on whether the pulsatile nature of GABA release from beta cells meaningfully regulates pulsatile insulin secretion. In support of this concept, manipulating GABA secretion with allylglycine and vigabatrin produces opposing effects on insulin secretion of over or under secretion, respectively, and both manipulations destabilize the periodicity of insulin secretion (12). Thus, the pulsatile pattern of GABA secretion and its impact on insulin secretion suggests that GABA is important for pacing islet activities.

## The effect of GABA on insulin secretion

In the early 1980s, researchers attempted to understand the islet GABA system through whole pancreas perfusion while others performed oral and intravenous administration of GABA or GABA receptor agonists and antagonists in humans and animals. Kawai and Unger performed one of the earliest experiments to show a definitive effect of GABA on islet hormone secretion by dynamic perfusion of whole dog pancreas (96). Their study showed that GABA (1-100 µM) robustly inhibited insulin secretion stimulated by 5.5 mM glucose. The inhibitory effect of GABA on insulin secretion was repeated in rat islets by Gu et al. in 1993 (97). In addition, the Baekkeskov group showed that overexpression of GAD65 in mouse beta cell, which greatly increased islet GABA content, robustly impaired first-phase insulin secretion and glucose responsiveness (98). Collectively, these results demonstrated that GABA is an autocrine inhibitory signal for insulin secretion. This data contrasts with the electrophysiology of the GABA_A_R, as explained below, where GABA can contribute to either beta cell depolarization or hyperpolarization depending on the experimental conditions. Classifying GABA as purely inhibitory or excitatory is an oversimplification of a complex underlying physiology.

## The function of GABA_A_ receptors in islet physiology

Ionotropic GABA_A_Rs are ligand-gated Cl^-^ channels that exert control over membrane potential and are widely distributed in the CNS. GABA_A_Rs consist of hetero-pentameric combinations of different subunits expressed from 19 genes categorized into different classes and subunit isoforms: α (1–6), β (1–4), γ (1–3), δ, ϵ, θ, π and ρ(1–3) (99). GABA_A_Rs most frequently consist of two α, two β and one γ subunit, with two GABA binding sites located at the β2+/α1- subunit interface. Although there are thousands of theoretical GABA_A_R assemblies, only select combinations create functional receptors *in vivo* (99). The different GABA_A_R subtypes exhibit marked difference in their localization (synaptic or extrasynaptic), Cl^-^ conductivity, sensitivity to GABA, time until desensitization, and pharmacological profile (99). Some GABA_A_R isoforms respond rapidly to high concentrations of GABA at the synapse and quickly deactivate/desensitize (phasic currents), while others are specialized for generating constant responses to ambient GABA and desensitize slowly or incompletely (tonic currents) (100). GABA_A_Rs are further subject to control through desensitization, deactivation, trafficking, membrane insertion and retrieval, and phosphorylation which dynamically adjust and/or maintain GABA sensitivity (101).

### GABA_A_R subunit expression in beta cells relates to tonic and phasic Cl^-^ conductances

GABA_A_R subunit expression directs the receptor localization and physiological properties. In neurons, the GABA_A_R α1, α2, and γ2 subunits impart preferential synaptic localization while the α4 and δ subunits are mainly extrasynaptic (100). Subunits α1 and α2 have the lowest affinity for GABA and desensitize quickly to efficiently respond to bursts of highly concentrated GABA at the synapse without being activated by background GABA. The α4, α5, and α6 subunits have a higher affinity for GABA, responding to low concentrations of ambient tonic GABA and desensitizing incompletely, enabling persistent, steady currents (99, 100). These designations are not absolute as all receptor subtypes are found extrasynaptically and synaptic receptors also mediate tonic responses depending on localization and cell type.

Functional GABA_A_Rs have been identified in primary beta cells at the mRNA, protein, and electrophysiological level (10, 76). In human beta cells, the α2, α5, β3, and γ2 subunits are expressed (10, 76) combining to form at least two functionally distinct GABA_A_Rs with different affinities for GABA, opening rates, and Cl^-^ conductances (10). These receptors may represent the low affinity α2β3γ2 and high affinity α5β3γ2 GABA_A_R subtypes (10). GABA_A_R subunit expression and responses shift affinity profile in human islets from donors with T2D (10), perhaps in response to the lower levels of islet GABA synthesis and secretion.

GABA_A_R subunits have also been detected in mouse islets, with the α4, β3, γ2, and δ subunits being prominent (10, 102). One report suggests the γ2 isoform, while possibly expressed in the other mouse islet endocrine cells, is not expressed in the mouse beta cells (10). These subunits suggest mouse beta cells are attuned to tonic GABA (α4β3δ). Expression of GABA_A_R receptor subunits has also been studied in rat islets with many different subunits detected that could comprise both tonic and phasic receptor subtypes (11, 102).

The subunit expression data indicate that beta cell GABA_A_R subtype, and thus physiological and pharmacological profile (sensitivity to modulatory drugs), differ significantly by species and disease state (10, 45). Experiments to systematically link GABA_A_R subunit expression to receptor behavior, particular to comprehend tonic and phasic GABA conductances, have not yet been undertaken in beta cells, although some clues are available. Measurements of rat and human beta cell native GABA_A_R responses to interstitial GABA (no external GABA applied) showed clear tonic conductances (11, 45), so it reasonably clear that beta cells have tonic GABA responses. Information on phasic GABA responses is more elusive as the best data available are from human beta cells transduced to overexpress the synaptic α1β1 GABA_A_R which showed both phasic and tonic responses (62, 76, 83). More exploration is needed to definitively determine the functional realities of the native GABA_A_R subunit composition in islets and link the specific subunits to tonic and phasic receptor behavior and overall islet function.

### GABA_A_R control over beta cell membrane potential

In the CNS, GABAergic inhibition is heavily mediated by the GABA_A_R and applying exogenous GABA to isolated islets tends to be inhibitory to insulin secretion at the macroscopic level. Thus, blocking GABA_A_R was the natural first approach to identify the mechanism of islet cell GABA signaling. Yet, when Kawai and Unger applied the potent GABA_A_R antagonist bicuculline, insulin secretion was inhibited rather than enhanced (96). The inhibition of insulin secretion by blocking GABA_A_R suggests a stimulatory effect of GABA_A_R channel opening. Decades later, Braun et al. provided additional evidence for the GABA stimulatory hypothesis by showing that the GABA_A_R antagonist SR-95531 also inhibited insulin secretion elicited by 6 mM glucose, concluding that GABA is an autocrine excitatory signal to beta cells. That GABA_A_R activation appears to be stimulatory contrasts with the finding that applied GABA is inhibitory to glucose-stimulated insulin secretion, an effect that has been repeatable across a wide range of studies spanning 40 years.

A stimulatory action of GABA_A_R in beta cells is the opposite of what occurs in most neurons where GABA_A_R is classically inhibitory. This is explained to be because beta cells, like immature neurons, maintain a high intracellular Cl^-^ concentration, [Cl^-^]_i_, of 34 mM (103), while mature adult neurons maintain a low [Cl^-^]_i_ at or below the Nernst equilibrium of 10 mM (104). The Cl^-^ electrochemical gradient determines the equilibrium potential for Cl^-^ currents (E_Cl_-). At membrane voltages below E_Cl_-, Cl^-^ flows out of the cell (inward current) and at membrane voltages above E_Cl_-, Cl^-^ flows into the cell (outward current) (105).

A seminal study on the function of GABA_A_R was conducted in human beta cells by Braun et al. using overexpression of GABA_A_R α1 and β1 subunits as a biosensor. Braun et al. showed that GABA application to resting beta cells in low glucose induced transient inward currents, characteristic of GABA_A_R-specific Cl^-^ currents that are depolarizing (76). These currents were sensitive to the GABA_A_R antagonist SR-95531 confirming the involvement of functional GABA_A_Rs (76). Such inward GABAergic currents contributed to depolarizing the beta cell when membrane potential is below E_Cl_- and even triggered single action potentials (76). However, to stimulate Ca^2+^ influx and hormone secretion, opening of GABA_A_Rs in low glucose would also need to overcome the strongly hyperpolarizing ATP-sensitive potassium channels (K_ATP_). Consistent with this notion, antagonizing GABA_A_R with SR-95531 reduced insulin secretion in 6 mM glucose (just above the threshold for electrical activity) but had no effect on insulin secretion at lower glucose levels (76).

**Figure 4:**
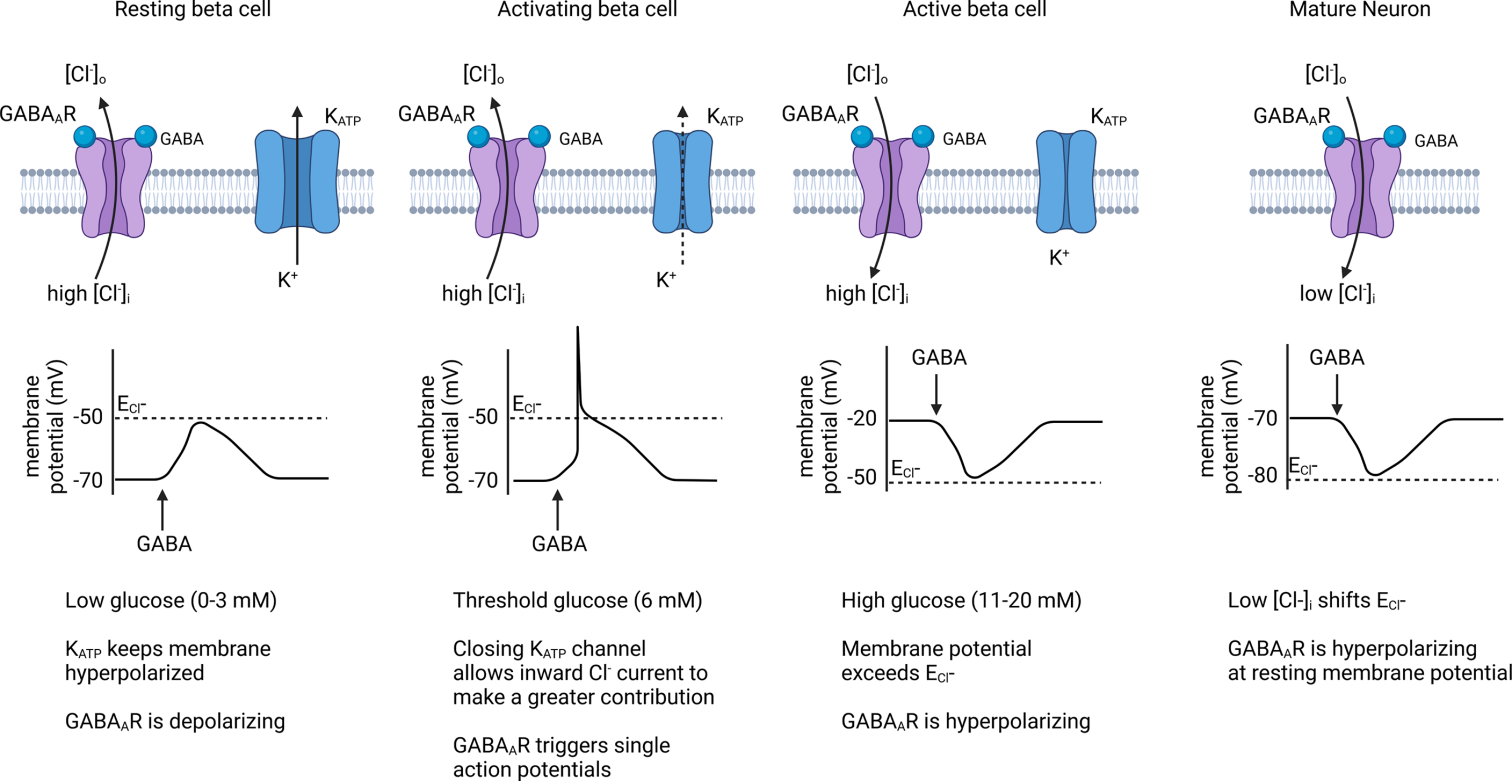
Effect of GABA_A_R chloride channels on beta cell membrane potential. When membrane potential is below the Cl^-^ equilibrium potential (E_Cl_-), endogenous GABA_A_R activation contributes to beta cell depolarization and can trigger action potentials in threshold-activated beta cells. Once the beta cell membrane becomes depolarized past E_Cl_- during an active phase, GABA_A_R activation results in repolarization of the membrane, causing an inhibitory effect on insulin secretion. Because beta cells have a high [Cl^-^]i, its sets the E_Cl_- at a higher level than for mature neurons where GABA_A_R is generally inhibitory. Created with BioRender.com.

Application of GABA at 6 mM glucose depolarizes beta cells from resting potential to approximately -55 mV (close to the E_Cl_-) and increases the frequency of action potentials at this threshold glucose concentration (76). However, high concentrations of GABA (e.g. >100 µM) will clamp the membrane potential to the equilibrium chloride potential (E_Cl_-) and prevent regenerative electrical activity (76). Thus, GABA applied at typical experimental concentrations (which may be supraphysiologic) induces a large Cl^-^ flux that clamps the membrane potential below the threshold for the opening of P/Q type Ca^2+^ channels (above -20 mV), the channels that are responsible for insulin granule exocytosis (106–108), which can explain the often observed reduction in insulin secretion despite GABA being depolarizing at sub-activating glucose. Furthermore, whenever membrane potential exceeds E_Cl_-, the direction of the GABA invoked Cl^-^ current changes direction to hyperpolarize the beta cell (109). Braun et al. reported the E_Cl_- in human beta cells to be approximately -50 mV, which results in a net inward (hyperpolarizing) driving force for Cl^-^ ions at 0 mV (76). The direction of GABAergic currents on membrane potential thus acutely depends on the beta cell activation state and experimental conditions including buffer electrolyte composition, glucose concentration, and the local concentration of GABA (**Figure 4**).

The general hypothesis that GABA is electrically excitatory in low glucose and inhibitory in high glucose was systematically tested and confirmed in INS-1 beta cells by Dong et al. who also measured that the reversal potential for GABA_A_R currents in INS-1 beta cells to be -43 mV, at the sweet spot between resting and activated membrane potentials (109). In accordance with the Goldman–Hodgkin–Katz solution of the Nernst-Plank equation, any significant ion conductance will push the membrane potential towards the equilibrium potential of that particular ion (110). Thus, GABA_A_R may act a bit like a voltage clamp in the islet, increasing beta cell excitability at resting conditions and moderating beta cell excitability in activating conditions (**Figure 4**).

Experiments to measure the average open probability and mean current of the native beta cell GABA_A_Rs at different membrane potentials showed beta cell GABA_A_Rs are outwardly rectifying (10, 11). This means that the beta cell GABA_A_R carries a low Cl^-^ current at hyperpolarized potentials but current increases as the membrane becomes more depolarized. Outward rectifier channels are closed (deactivated) at negative membrane potentials and progressively activate with membrane depolarization. Outward rectification of the GABA_A_R could theoretically increase beta cell sensitivity to GABA upon beta cell depolarization independently of GABA secretion rate.

There are several additional important questions that arise surrounding GABA_A_R based on factors that are understood in neurons but have been understudied in beta cells. These include: 1) Do beta cells exert control over GABA sensitivity by GABA_A_R internalization and cell surface trafficking? (111); 2) What are the physiological implications of different GABA_A_R subtypes in the islet? (112); 3) Like neurons, do beta cells exhibit a developmental GABA switch due to changes in [Cl^-^]_i_ that track with maturation? (113); 4) To what extend do beta cells use “shunting inhibition” where tonic GABAergic chloride conductance is initially excitatory but further increasing conductance overpowers the excitation and results in inhibition? (114); 5) What are the relative contributions of phasic and tonic currents to GABA signaling in the islet? (100); and 6) Do beta cells experience “off responses,” where the sudden withdrawal of GABA generates a rebound excitation response? (115, 116).

## The function of GABA_B_ receptors in islet physiology

The mechanism by which applied GABA inhibits insulin secretion may also involve GABA_B_Rs in beta cells (117–120). GABA_B_Rs are heterodimers formed from subunits GABBR1 and GABBR2 (121) to form an inhibitory G protein-coupled receptor (G_i/o_-GPCR). The family of G_i/o_-GPCRs in beta cells includes the somatostatin receptors, which function by stimulating the opening of hyperpolarizing G protein-activated inward rectified potassium channels (GIRK), reducing the activity of adenylyl cyclase, which results in lower cyclic AMP (cAMP), and inhibiting voltage gated Ca^2+^ channels (VGCC) (122, 123). In this manner, GABA_B_R signaling is proposed to activate similar inhibitory mechanisms as somatostatin receptor signaling. Selective induction of G_i_-GPCRs in beta cells also activates Na^+^/K^+^ ATPases (NKAs) and initiates islet Ca^2+^ oscillations, further suggesting that GABA could be important for tuning pulsatile insulin secretion (124). Studies investigating the role of the GABA_B_R using agonists and antagonists demonstrate that GABA_B_R activation reduces insulin secretion (45, 118, 120). An inhibitory interaction between G_i/o_ and N-, P/Q-, and R-type channels is one of the classical pathways of GABA_B_R VGCC channel regulation in neurons (125). However, calcium flux within the islet does not acutely change upon addition of baclofen, a selective GABA_B_R agonist (118), thus GABA_B_R may mainly act in beta cells *via* reducing cAMP and opening GIRKs.

Some studies indicate that human beta cells do not form functional GABA_B_R complexes under basal conditions. This is because human beta cells only express detectable levels of one of the GABA_B_R obligatory subunits, GABBR1, while the second subunit, GABBR2, is below the detection threshold (10, 120). Mouse and rat beta cells, on the other hand, express both obligatory GABBR1 and GABBR2 subunits and have functional GABA_B_Rs that contribute to insulin secretion and glucose homeostasis (118, 119, 126). Interestingly, GABBR2 expression in human islets is inducible by cAMP signaling. Multiple strategies to raise levels of cAMP (e.g. forskolin, exendin4, 8Br-cAMP) each robustly increased *GABBR2* mRNA levels, resulting in formation of functional GABA_B_R units that exert negative control over insulin secretion (120). The levels of cAMP in beta cells are influenced by incretins like glucagon-like peptide-1 (GLP-1) (127). Thus, at least in human islets, GABBR2 expression may be held in reserve unless a period of prolonged and elevated cAMP necessitates GABA_B_R inhibitory control to bring beta cell cAMP and electrical activity back to basal levels.

## GABA as a mediator of the paracrine cross-talk between pancreatic islet cells

The pancreatic islet is a dense cellular cluster arranged as an endocrine micro-organ formed by several cell types that control glucose metabolism, intermediary metabolism, and food ingestion. One of the most important processes that allows the pancreatic islets to be the master regulator of glycemia is the paracrine relation between their cells. In a nutrient abundance period, beta cells secret the hypoglycemic hormone insulin that inhibits the secretion of the hyperglycemic hormone glucagon by the pancreatic alpha cells. On the other hand, to counterattack the fasting state the alpha cells secrete glucagon, which stimulates insulin secretion. The secretion of both insulin and glucagon is inhibited by the delta cell-secreted somatostatin. In consonance with the hormones, other islet-released molecules, such as GABA, form an intricate paracrine network that controls the hormone release within the islet and, consequently, controls glucose homeostasis.

## Effect of GABA on glucagon secretion

The main alpha cell product is glucagon, a 29 amino acid peptide hormone known as the major hypoglycemia-counteracting agent. The glucagon hyperglycemic effect occurs mainly in the liver, where, after binding to its G protein-coupled receptor (GCGR) (128), glucagon promotes hepatic glucose output stimulating gluconeogenesis and glycogenolysis (129). The key factor in glucagon secretion is blood glucose levels. Unlike beta cells, low circulating glucose leads to an increase in glucagon secretion. In a mechanism that involves T-type Ca^2+^ channel opening followed by membrane depolarization and Ca^2+^ and Na^+^ influx, the alpha cells secrete glucagon during a glucose-privation period (130, 131). In addition to the glucose stimulus, glucagon secretion is regulated by other circulating molecules, including amino acids (132) and free fatty acids (133) and by paracrine effectors, such as insulin (134) and GABA (135). Multiple studies have shown that GABA negatively regulates glucagon release (46, 102, 135), helping to refine the pancreatic response to alteration in the blood glucose.

The GABA mediation over the islet cell network depends on its binding to GABA receptors. While the ionotropic GABA_A_R was detected in the rodent (23, 82) and human (45) alpha cell membrane surface, GABA_B_R was detected only in rat alpha cells (62). The GABA_A_R is likely important in alpha cell responses where the alteration in the membrane potentiation suppresses the alpha cells’ electric activity by making the potential more negative and more unlikely to generate an action potential, thus suppressing glucagon release (135).

In glucose homeostasis, where insulin and glucagon antagonize each other, the GABAergic system appears to act as a counterbalance. During the postprandial period, a rise in the blood glucose concentration is observed, which induces the beta cell secretion of insulin and GABA. In this nutrient abundance period, where there is no need to mobilize endogenous energy stores, no glucagon is necessary. This information is conveyed to alpha cells through insulin and GABA signaling. A complication of this feedback mechanism is that macroscopic GABA release from beta cells does not increase along with insulin in response to glucose stimulation. This discrepancy is resolved by data showing that insulin enhances GABA_A_R activity in alpha cells *via* increased membrane translocation of the intracellular receptor pool (46), thereby increasing responsiveness to available GABA despite GABA release not being coregulated with insulin. Thus, islet endocrine cells can be envisioned to tune their sensitivity to ambient GABA as a means of controlling excitation rather than the islet interstitial GABA levels changing dramatically in response to glucose or other stimuli. That GABA reinforces the inhibitory communication from beta to alpha cells suggests the islet GABA system as a target to treat diabetes. Dysregulation of alpha cell glucagon secretion in low glucose contributes to diabetic hyperglycemia and is observed in T2D, T1D and pre-T1D autoantibody positive individuals (136, 137). Coincidentally, islets from donors with T2D and T1D are also observed to be depleted of GABA (12). These results ask us to consider whether the loss of islet GABA in diabetes is responsible for poor control over glucagon secretion. Could pharmacologically targeting GABA signaling improve glycemia in diabetic patients?

## Effect of GABA on delta cells

Delta cells are the third most abundant cell type in the pancreatic islets and, in a paracrine fashion, inhibit insulin and glucagon secretion. There is evidence that beta cells modulate somatostatin (SST) secretion through Urocortin3 signaling (138). Moreover, delta cells also express GABA receptors, indicating that beta cells could modulate delta cells through GABA signaling (76). However, studies of GABA on delta cells are still very scarce. There is very little data using sophisticated modern techniques to convincingly demonstrate how GABA modulation affects SST secretion.

Most studies developed with GABA and delta cells are focused on GABA_A_R signaling. There is not much information on the importance of GABA_B_Rs in delta cells. One study, Braun et al., 2004 demonstrated that the incubation of isolated rat pancreatic islets with CGP55845, a GABA_B_R antagonist, did not alter glucose-stimulated somatostatin secretion (118). On the other hand, it is reported that delta cells express high levels of GABA_A_R (76). Nevertheless, the known effects of GABA on pancreatic delta cells are controversial. Results from Braun et al. showed that the inhibition of GABA_A_R using an antagonist (SR-95531) reduces SST secretion in both low (3 mM) and high glucose (20 mM). In addition, in the same study, they demonstrated by patch-clamp experiments that incubation with GABA strongly depolarizes delta cells membrane potential, indicating that GABA secreted by beta cells could stimulate SST secretion (76). Other studies performed using static incubation showed that GABA did not change SST secretion (83, 135, 139). However, SST secretion stimulated by arginine was increased by addition of 100 μM of GABA_A_R antagonist bicuculline (135).

Consistent with the results of Braun et al. that GABA is stimulatory to delta cells, a study from the 1980s performed with perfused dog’s pancreas showed that GABA transiently increased SST secretion, and this stimulation was dose dependent. GABA increased SST secretion rapidly but desensitized within 1 min. When incubated with bicuculline (a GABA_A_R antagonist) in the presence of GABA, there was no alteration in SST secretion. Yet, when bicuculline infusion was discontinued, a quickly increased SST release was observed (96).

Differently, Robbins et al. demonstrated that in the perfused rat’s pancreas, incubation with muscimol, a GABA_A_R agonist, reduced glucose-stimulated SST secretion. This inhibition of SST secretion was not reversed when muscimol incubation was removed (140). Furthermore, consistent with the notion that GABA could inhibit SST secretion, a study demonstrated that a patient that presented somatostatinoma of the pancreas and high-level plasma SST received for 6 days sodium valproate, an elevator of GABA synthesis and secretion, presented an increased plasma level GABA leading a reduced plasma SST (141). Thus, further studies are needed to clarify the direction and extent of GABA’s effects on delta cell SST secretion.

It has been shown that GABA localizes differently in delta cells from beta cells. GABA in human delta cells is localized in vesicles but is cytosolic in beta cells due to VGAT being expressed in human delta cells but not in most beta cells (12). Despite having observed the expression of GAD65 in the human delta cells, there is still no evidence that delta cells synthesize GABA, particularly because removal of beta cells by STZ or T1D results in islets devoid of GABA (12). However, Gilon & Remacle demonstrated that a subset of delta cells from a rat’s pancreas selectively uptake radiolabeled GABA (43). In this sense, delta cells in the islet may function in a manner analogous to astrocytes that take up and clear excess GABA in the brain. Studies should be directed to understand if delta cell sequestered GABA is metabolized or re-secreted to modulate hormone secretion in an auto- or paracrine fashion.

## Open questions about the role of GABA in islets

Several open questions and discovery opportunities exist related to the role of GABA in the islet. First, the mechanism that controls basal secretion of GABA from the islet needs further clarification. Although GABA has been historically localized in synaptic-like microvesicles and large dense core vesicles (55, 62, 76, 83), the amount of secretion of GABA from these vesicles cannot account for the large background rate of release from beta cells that is unregulated by glucose (12, 77, 142). Additionally, studies have shown immunogold and immunohistochemical labeling of some vesicular GABA (62, 76), but uniformly diffuse, cytosolic GABA appears to be more dominant (12). It seems likely, therefore, that a mechanism for cytosolic secretion of GABA must be driving basal GABA secretion. VRAC was identified as a mechanism of non-vesicular secretion from the cytosol (12), but the physiological trigger that gates the isoform of VRAC permeable to GABA, particularly one that could account for pulsatile GABA release, is unknown. Real-time optical indicators of local endogenous GABA such as iGABASnFR (143) could be implemented to further elucidate endogenous triggers and dynamics of islet GABA secretion.

Next, given the recent identification of GABA as a regenerative factor for the islet, the role of GABA in pancreatic development and morphology has not been investigated. While mechanisms relating GABA signaling in alpha cell or ductal cell transdifferentiation to beta cells have been debated (22, 28, 29, 144), it would be interesting to understand whether GABA is important to beta cell maturation, islet development and morphogenesis, and whether there is a developmental switch in the direction of GABA_A_R currents on beta cell membrane potential like the one that exists in the CNS (113).

The complete mechanism to describe action of GABA on total islet endocrine cell function has not been adequately described. Multiple results showing both excitatory action on beta cell membrane potential (76, 145) and inhibitory effects on insulin secretion (12, 96) have been reported. However, the majority of these studies have utilized applied GABA. A confounding factor is that applied GABA or drugs that manipulate GABA receptors occur on top of endogenous beta cell GABA secretion and concentrations used may be supraphysiologic. Given the variability in methods used and difficulty in performing and interpreting the experiments due to the complex interactions between GABA_A_R, GABA_B_R, GABA metabolism and indirect signals such as potential GABA stimulation of SST secretion, the integrated effect of endogenous beta cell GABA signaling on insulin secretion is still not completely certain. Furthermore, the effect of GABA on the delta cell has been understudied. We propose that the majority of the evidence gathered thus far converges on endogenous beta cell GABA inhibiting insulin secretion in excited beta cells. Given the pulsatile nature of its efflux pattern, GABA may also be important for regulating the oscillatory rhythm of insulin secretion. Ultimately, a mouse strain with conditional knockout of the GAD enzymes and each type of GABA receptor in beta cells would be useful to confirm the role and importance of GABA in islet physiology.

Finally, the importance of GABA in diabetes pathology has yet to be clarified. Islet GABA levels are observed to be significantly decreased in T1D and T2D (10, 12, 45), but the cause of this decline in GABA is unknown. Because GABA appears to be a negative immune regulator (13, 14, 33, 146), does a loss of islet GABA make islets more susceptible to immune insult or contribute to triggering of autoimmunity? Once diabetes manifests clinically and most beta cells are lost or senescent, does a lack of GABA signaling from beta cells contribute to alpha and delta cell dysfunction? The role of this loss of GABA in the pathophysiology of diabetes has not been determined. On top of that, the biosynthetic enzyme GAD65 is one of the major autoantigens for T1D. GAD65 is expressed in alpha and delta cells yet the enzyme does not appear to function for GABA biosynthesis in these cell types (12). If alpha and delta cells do express GAD65, why are these cell types not targeted for destruction in T1D? While these questions remain to be addressed, it is clear that we must better understand how GABA influences the islet dysfunction that accompanies the major forms of diabetes.

**Figure 5:**
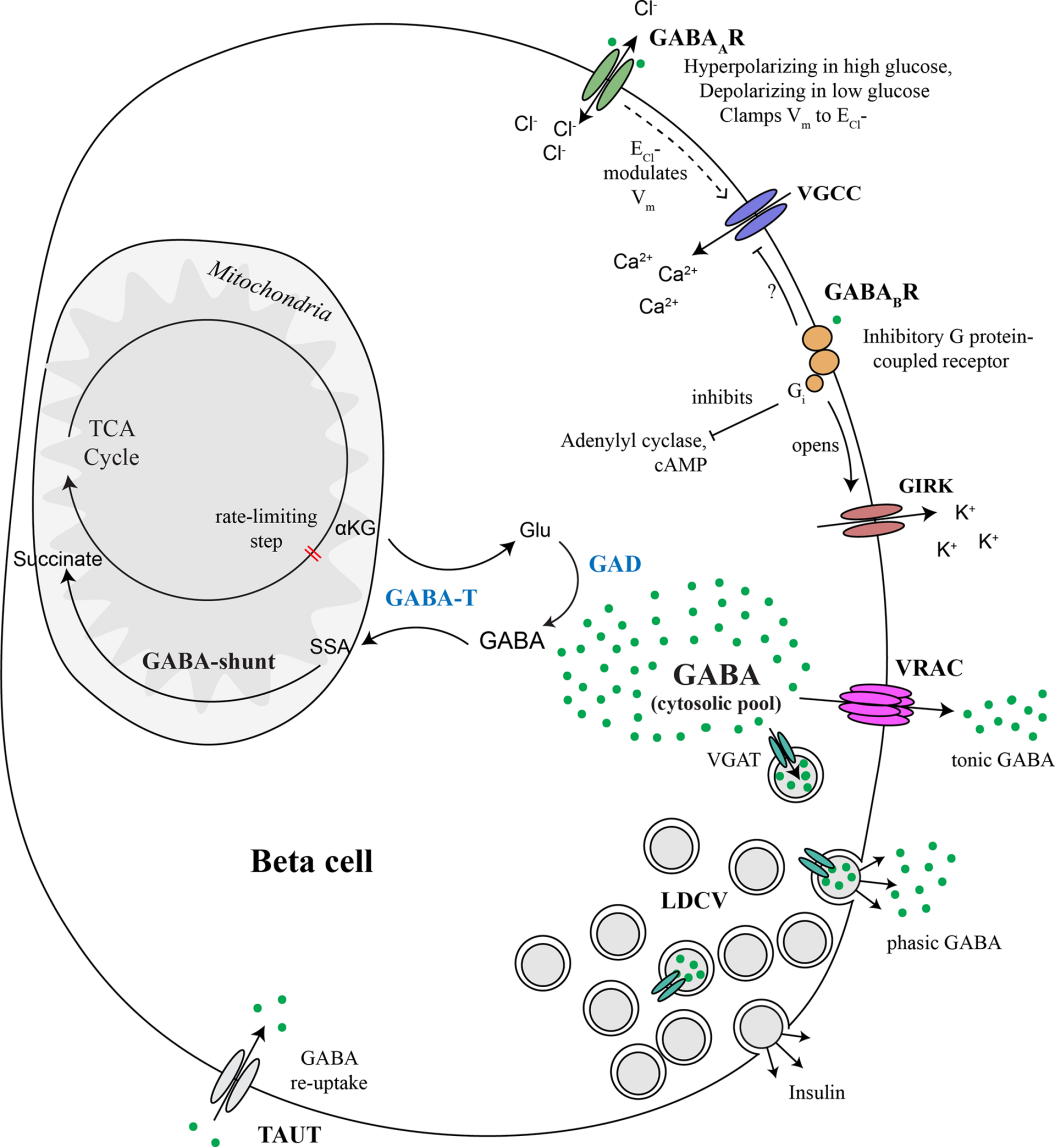
Major components of the beta cell GABA system. GABA is synthesized in the cytosol from glutamic acid (Glu) by the enzyme glutamic acid decarboxylase (GAD), of which there are two isoforms, GAD65 and GAD67. GABA is transaminated with alpha ketoglutarate (αKG) *via* GABA transaminase (GABA-T) to form glutamic acid (Glu) and succinate semialdehyde (SSA). SSA is in turn oxidized to form succinate, which enters the TCA cycle, thus connecting the GABA pool to beta cell metabolism. GABA is secreted to the extracellular space *via* volume-regulated anion channels (VRAC), which are a mechanism for non-vesicular secretion of osmolytes GABA and taurine from the cytosolic pool in response to changes in osmolarity or other triggers. This non-vesicular form of GABA release does not appear to be regulated by glucose. GABA is also be secreted from a sub-population of large dense core vesicles (LDCV) together with insulin. Vesicular packaging of GABA depends on the presence of the vesicular GABA transporter (VGAT), which is expressed in only a subpopulation of beta cells. Alternatively, we speculate that VMAT2 could serve as a vesicular GABA transporter in the absence of VGAT, although experimental evidence has not yet been generated to support this concept. Once secreted, interstitial islet GABA ligates GABA_A_ receptors (GABA_A_R), which are Cl^-^ channels. Chloride currents through opened GABA_A_Rs modulate membrane potential (V_m_) and thus control beta cell excitability. The direction that GABA_A_R pushes V_m_ depends on whether the V_m_ is presently above or below the equilibrium chloride potential (E_Cl_-). GABA_A_R activation can inhibit insulin secretion by clamping V_m_ to the E_Cl_- or hyperpolarizing the membrane back toward E_Cl_- when V_m_ is more electropositive in excited beta cells. GABA_A_R can contribute to beta cell depolarization when glucose concentrations are low and V_m_ is negative of E_Cl_-. GABA also ligates GABA_B_ receptors (GABA_B_R), which are inhibitory G protein (Gi)-coupled receptors that stimulate the opening of G protein-coupled inwardly-rectifying potassium channels (GIRKs) and inhibit adenylyl cyclase. Gi is also known to inhibit P/Q-type and N-type and Ca^2+^ channels but it is not yet confirmed whether this mechanism occurs in beta cells. Overall, the pancreatic islet integrates metabolic, ionotropic, and metabotropic signals together with the paracrine effects of GABA *via* responses invoked in alpha and delta cells, to result in a net effect of GABA on islet function.

In summary, this review comprises an effort to clarify a central model of the endogenous GABA system within the islet (**Figure 5**). Following the history of investigation of GABA in the islet has allowed us to identify future directions for the role of GABA in the islet and suggest the development of new tools to answer those questions.

## Author contributions

DH, SF, GS, and EP all contributed to writing the review. All authors commented and provided feedback on initial drafts. EP edited and completed the final draft of the manuscript.

## Funding

Funding for this research was provided by the NIH grant #R01DK124267 and the Diabetes Research Connection, Project Number 47.

## Acknowledgments

Thanks to Patrik Rorsman for providing example traces of tonic and phasic GABA_A_R Cl^-^ currents in human beta cells.

## Conflict of interest

The authors declare that the research was conducted in the absence of any commercial or financial relationships that could be construed as a potential conflict of interest.

## Publisher’s note

All claims expressed in this article are solely those of the authors and do not necessarily represent those of their affiliated organizations, or those of the publisher, the editors and the reviewers. Any product that may be evaluated in this article, or claim that may be made by its manufacturer, is not guaranteed or endorsed by the publisher.
