# Erratum: The role of GABA in islet function

**DOI:** 10.3389/fendo.2023.1301830

**Published:** 2023-10-02

**Authors:** Frontiers Production Office

**Affiliations:** Frontiers Media SA, Lausanne, Switzerland

**Keywords:** γ-Aminobutyric acid (GABA), islet, pancreas, signaling, receptor, insulin, beta cell

Due to a production error, there was a mistake in the penultimate sentence of the caption of **Figure 1**. The subscript “_A_” in “GABA_A_R” should have been “_B_”. The corrected caption appears below, along with **Figure 1**.

**Figure 1:**
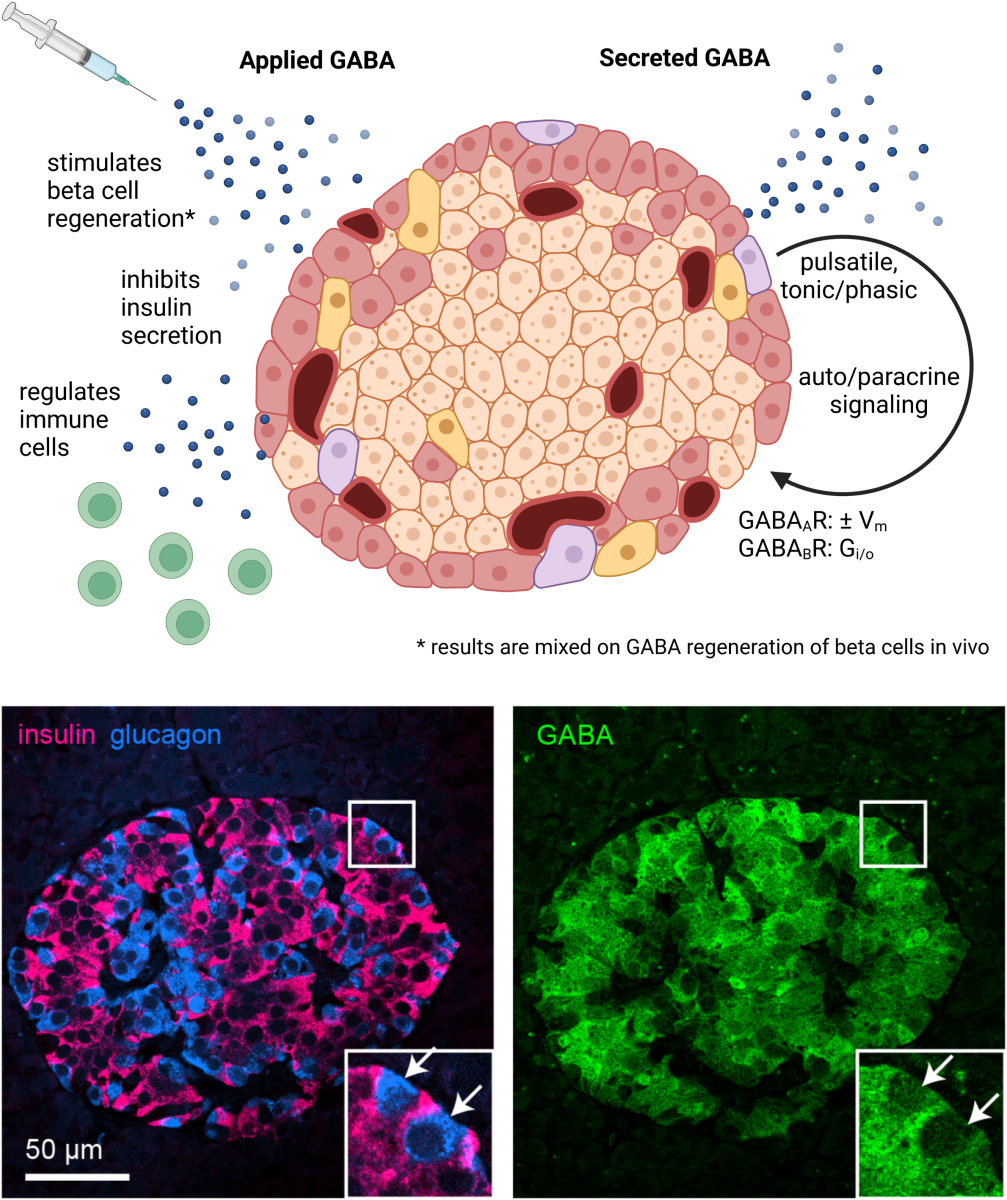
GABA in the whole islet. Application of exogenous GABA has various effects on the islet including stimulation of beta cell regeneration, inhibition of insulin secretion, and negative regulation of immune cells. Endogenous GABA levels are highly enriched in the islet, as high as in the brain, and GABA is synthesized in and secreted from the beta cells. Immunofluorescence image depicts a human islet. GABA is secreted via multiple pathways that are both regulated and unregulated by glucose and with pulsatile, tonic, or phasic dynamics. Once secreted, GABA acts *via* GABA_A_R ligand-gated chloride channels and GABA_A_R inhibitory G protein coupled receptors. Set by the chloride equilibrium potential, in beta cells GABA_A_R signaling can be excitatory in low glucose and inhibitory in high glucose, while in alpha cells GABA_A_R signaling is inhibitory. GABA_B_R signaling is also inhibitory but may only be active in mouse and not human beta cells under typical physiological conditions. Created with BioRender.com.

The publisher apologizes for this mistake. The original version of this article has been updated.

